# Regenerative Trifecta: A Multimodal Regenerative Approach to Soft Tissue Injury Recovery and Return to Sport

**DOI:** 10.7759/cureus.114620

**Published:** 2026-08-16

**Authors:** David Greene

**Affiliations:** 1 Research and Development, R3 Stem Cell, Scottsdale, USA

**Keywords:** exosomes, mesenchymal stem cells, nba injury trend, orthobiologics, platelet-rich plasma, regenerative medicine, return to sport, soft tissue repair, sports injuries, uc-msc

## Abstract

Soft tissue injuries - affecting tendons, ligaments, and muscles - are the leading cause of missed games, lost seasons, and early career endings in competitive sports. Injury rates across major professional leagues have risen significantly over the past decade, with the knee, groin, hip, and thigh bearing the greatest burden. The financial impact is enormous at the high school and collegiate levels alone, while at the professional level, soft tissue failures have decided playoff outcomes and derailed championship-caliber teams.

Standard treatments - rest, physical therapy, nonsteroidal anti-inflammatory drugs (NSAIDs), and corticosteroids - address symptoms but not the underlying biology. Tendons and ligaments are poorly vascularized structures that heal through fibrotic scar tissue rather than true collagen restoration, leaving repaired tissue mechanically weak and prone to re-injury.

Regenerative medicine offers a fundamentally different approach. Mesenchymal stem cells (MSCs), platelet-rich plasma (PRP), and extracellular vesicles (EVs) actively remodel the injury environment, promoting improved healing quality and reduced scar formation compared to standard care. Clinical evidence from randomized controlled trials and meta-analyses demonstrates that PRP accelerates return-to-sport timelines in acute muscle injuries , while preclinical and early translational evidence supports MSC and EV therapy for improving healing quality.

This paper reviews the evidence for regenerative biologics in sports medicine and presents the Regenerative Trifecta protocol developed by R3 Stem Cell, one of the world's largest multinational regenerative networks. It is a coordinated combination of umbilical cord mesenchymal stem cells (UC-MSCs), PRP, and exosomes designed to address the full biology of soft tissue healing. The mechanism of each component is described along with the clinical data supporting its use, and the rationale for multi-modal over single-modality treatment, with particular attention to elite and masters athlete populations where healing biology and clinical need converge most critically.

## Introduction and background

Soft tissue injuries

Soft tissue injuries involving tendons, ligaments, muscles, and their insertion zones are the defining medical challenge in competitive athletics at every level of participation. Across professional leagues, they account for the majority of missed games, derailed seasons, and premature career endings. The epidemiological burden is substantial and growing. A comprehensive cross-sport analysis of Major League Baseball (MLB), the National Basketball Association (NBA), the National Football League (NFL), and the National Hockey League (NHL) documented a mean of 62.49 injury events per 100 players per season over a 13-season observation period, with a statistically significant 5.5% increase in injury incidence between 2007 and 2019. The knee, groin, hip, and thigh were the anatomical regions most consistently affected [1-3].

The economic consequences are considerable at every competitive level. At the professional level, a single season-ending soft tissue injury eliminates franchise-defining athletes at moments of maximum competitive importance - a reality rendered viscerally clear by the cascade of hamstring, Achilles, and calf injuries that defined the 2024-25 and 2025-26 NBA playoff cycles. The Denver Nuggets' playoff elimination in the 2025-26 season was significantly impacted by soft tissue injuries to two key players - Aaron Gordon and Peyton Watson - whose injuries exemplify the clinical challenge of incomplete soft tissue healing in high-performance athletes. In the most recent 2026 NBA season, the Los Angeles Lakers succumbed early in the playoffs, with one of their star players, Luka Doncic, unable to return from a lingering soft tissue injury to his hamstring.

Standard care for soft tissue injuries - rest, physical therapy, nonsteroidal anti-inflammatory drugs (NSAIDs), and corticosteroid injection - manages symptoms but does not address the fundamental biology of why tendons and ligaments heal poorly. These structures are hypovascular, receiving their blood supply from sparse peritendinous and intratendinous vessels insufficient to sustain the inflammatory and proliferative demands of robust repair. When injured, they heal via fibrotic scar formation rather than functional collagen restoration, resulting in mechanically inferior tissue that remains vulnerable to reinjury under athletic loading conditions [4-8].

Regenerative medicine offers a fundamentally different approach. Mesenchymal stem cells (MSCs), platelet-rich plasma (PRP), and extracellular vesicles (EVs) actively remodel the injury environment, promoting improved healing quality and reduced scar formation compared to standard care. Clinical evidence from randomized controlled trials and meta-analyses demonstrates that PRP accelerates return-to-sport (RTS) timelines in acute muscle injuries, while preclinical and early translational evidence supports MSC and EV therapy for improving healing quality [1,2,9-11].

It is worth noting at the outset that regenerative and biologic medicine in musculoskeletal practice extends well beyond PRP, MSCs, and exosomes, and that these three modalities do not represent the full range of options available to patients and clinicians. Lower-cost, more widely accessible interventions - including dextrose prolotherapy, ultrasound-guided hydrodissection of adherent or entrapped soft tissue and peripheral nerves, and extracorporeal shockwave therapy (ESWT) - have an established evidence base in tendinopathy and related conditions and are commonly used before, alongside, or instead of higher-cost biologic therapies, depending on the lesion type, chronicity, patient age, and treatment goals [12-14].

These modalities are meaningfully less expensive and more widely available than cellular or PRP-based therapies, and for many patients they represent an appropriate first-line or adjunctive option rather than a step to be bypassed on the way to more advanced biologics. This paper focuses specifically on the Regenerative Trifecta protocol described below and does not attempt a comprehensive review of the full regenerative and orthobiologic landscape; readers evaluating treatment options for an individual patient should weigh the complete range of available interventions, not solely those reviewed in depth here.

A related consideration, often underappreciated in the biologics literature, is that soft tissue healing is shaped not only by local, tissue-level factors but by the patient's broader metabolic and endocrine status. Prediabetes and diabetes are associated with impaired tendon structure and healing capacity independent of local injury factors, with emerging evidence implicating circulating advanced glycation end-products as a driver of collagen disorganization and delayed repair even when glycemic control is otherwise adequate [15]. Vitamin D deficiency, which is highly prevalent in the general population, has similarly been associated with poorer postoperative tendon healing and higher retear rates [16].

Dyslipidemia, nutritional insufficiency, and hormonal imbalance - including thyroid and sex hormone status - are increasingly recognized as potential contributors to impaired tissue repair, though the clinical evidence in this domain remains earlier-stage than that for PRP or MSC therapy. For clinicians, the practical implication is that a patient who responds poorly to a regenerative procedure, biologic or otherwise, may have an unrecognized systemic or metabolic contributor to impaired healing that warrants evaluation before the outcome is attributed to the procedure itself. Screening for these factors, where clinically indicated, is a reasonable component of a comprehensive treatment plan for patients with persistent or refractory soft tissue injury.

This paper reviews the evidence base for regenerative biologics in sports medicine and describes a proprietary Regenerative Trifecta protocol. Devised by one of the world's largest regenerative therapy networks, R3 Stem Cell, the protocol combines umbilical cord-derived mesenchymal stem cells (UC-MSCs), PRP, and exosomes in a coordinated treatment strategy that addresses the full spectrum of soft tissue healing biology. The mechanisms underpinning each component are reviewed along with the clinical evidence supporting their use, and the rationale for multi-modal combination over any single-modality approach. The application of this protocol to recreational athlete populations is discussed, where the clinical need and the biology of impaired healing intersect most acutely [1].

## Review

Biology of incomplete soft tissue healing

Vascular Architecture and Intrinsic Repair Capacity

The fundamental reason soft tissue injuries recur at high rates lies primarily in the biology of the tissue itself, compounded by rehabilitation quality, RTS timing, and biomechanical factors. Tendons and ligaments are predominantly hypovascular structures composed of densely organized collagen I fibers maintained by metabolically quiescent tenocytes and fibroblasts. Their blood supply derives from peritendinous paratenon vessels, insertional vascularity at the osteotendinous junction, and sparse intratendinous vessels - a vascular architecture that provides sufficient metabolic support under basal conditions but is wholly inadequate to sustain the oxygen delivery, immune cell recruitment, and growth factor flux required for robust inflammatory and proliferative healing responses [7,8].

Cartilage, at the fibrocartilaginous interface between tendon and bone, presents an even more extreme vascular deficit. Benjamin and Ralphs characterized the biology of fibrocartilage cells in insertional anatomy, establishing that the poorest healing capacity in the musculoskeletal system is concentrated precisely at the enthesis - the zone where the highest mechanical forces are applied during sport and where clinical failures are most catastrophic [8].

Three-Phase Healing Cascade and Where It Fails

Soft tissue healing proceeds through three overlapping phases: inflammatory (days 1-7), proliferative (days 7-21), and remodeling (weeks 3-52). Each transition requires specific cellular signals and growth factor concentrations that are reliably available in well-vascularized tissues but consistently insufficient in tendon and ligament [4,6].

In the inflammatory phase, pro-inflammatory M1 macrophages clear cellular debris but also elaborate proteolytic matrix metalloproteinases (MMPs) that degrade extracellular matrix proteins. The critical transition to the proliferative phase requires a shift from M1 to the pro-regenerative M2 macrophage phenotype - a polarization event driven by specific cytokines and paracrine signals, most prominently interleukin-10 (IL-10), interleukin-4 (IL-4), and MSC-derived factors. In the absence of an adequate paracrine microenvironment, this transition fails or is incomplete, resulting in prolonged M1 dominance, excessive proteolytic activity, and disordered collagen deposition [9,10].

Macrophages are immune cells that show up at an injury site and switch between two functional states depending on what the tissue needs - that's the M1/M2 framework.

M1 (pro-inflammatory) phenotype are the *first responders*. M1 macrophages dominate the early inflammatory phase (roughly days 1-7 after injury). Their job is cleanup: they clear cellular debris, dead tissue, and pathogens, and they do this partly by releasing MMPs - enzymes that break down damaged extracellular matrix. This is necessary, but M1 activity is destructive by nature. If it persists too long, it keeps degrading tissue faster than it can be rebuilt.

M2 (pro-regenerative) phenotype macrophages are the *builders*. They secrete anti-inflammatory and pro-repair signals - including growth factors and cytokines like IL-10 - that shift the tissue environment from breakdown mode to rebuild mode. This is when fibroblasts proliferate, new collagen gets laid down, and blood vessels start to form.

Healing is not about having M2 cells instead of M1 - it's about the timing of the switch from one to the other. If the M1-to-M2 transition happens on schedule, you get organized collagen I deposition (strong, functional tissue). If the transition is delayed or incomplete - which happens more often in hypovascular tissue like tendons and ligaments, or in older patients with reduced paracrine signaling - M1 activity lingers too long. The result is excess proteolytic breakdown and disorganized collagen III deposition: fibrotic scar tissue that's mechanically weaker and more prone to re-injury.

This is why MSCs matter so much mechanistically: they're one of the main drivers of that M1→M2 switch, acting as a kind of *traffic signal* that tells the immune response when to stop demolishing and start rebuilding.

The consequence of not having the *traffic signal* is the characteristic biology of soft tissue injury repair: predominantly collagen III deposition (versus the load-bearing collagen I of healthy tendon), disordered fiber alignment, reduced tensile strength, and scar tissue formation that lacks the mechanical properties of native tendon matrix [4,6]. In extreme cases of dysregulated healing, the same fibrotic pathways can lead to heterotopic ossification - the formation of bone in soft tissues-demonstrating the critical importance of precise biological signaling in guiding repair toward functional tissue rather than pathological mineralization. Athletes who are supposedly fully recovered under standard care frequently RTS with tissue that is structurally inferior to what was present before injury - explaining why re-injury rates within the same season remain 12%-18% in professional sports populations, and why recurrence risk persists for multiple seasons after an initial injury [1,2].

Macrophage Polarization as the Central Regulatory Event

The M1-to-M2 macrophage transition is increasingly recognized as the single most therapeutically important biological event in soft tissue healing. Bernardo and Fibbe established that mesenchymal stromal cells function as sensors and switchers of inflammation - responding to inflammatory signals with the secretion of anti-inflammatory cytokines that drive M2 polarization and establish the conditions for proliferative repair. Uccelli et al. similarly demonstrated that MSCs modulate innate and adaptive immunity through mechanisms including paracrine cytokine secretion, direct cell contact-mediated immunosuppression, and induction of regulatory T cells [9,10].

Caplan has argued persuasively that MSCs function not primarily as differentiation-competent stem cells but as "drugstores," site-regulated dispensaries of bioactive molecules that sense the injury microenvironment and secrete the precise combination of factors required to convert it from a pro-inflammatory to a pro-regenerative state. Caplan et al.'s 2011 description of MSCs as injury drugstores, and their 2016 reconceptualization of MSCs as Medicinal Signaling Cells that act as *sentinels and safeguards* of injured tissue, define the mechanistic framework on which the Trifecta protocol is built [2,3].

Three Deficits Driving Poor Repair

R3 Stem Cell identifies three core biological deficits that determine whether a soft tissue injury will heal functionally or fibrotically:

Deficit 1: Insufficient paracrine signaling: Injured tissue lacks the MSC-derived cytokines and bioactive molecules needed to drive M1-to-M2 macrophage polarization and establish a regenerative microenvironment. This deficit is most pronounced in aged athletes whose endogenous MSC populations are reduced in both number and paracrine potency.

Deficit 2 (Inadequate growth factor delivery): The hypovascular nature of tendons and ligaments limits the endogenous delivery of platelet-derived growth factor (PDGF), transforming growth factor-β (TGF-β), vascular endothelial growth factor (VEGF), and insulin-like growth factor-1 (IGF-1), molecules essential for fibroblast proliferation, collagen synthesis, and neovascularization. Without exogenous growth factor supplementation, the proliferative phase of healing is attenuated and delayed.

Deficit 3: Pro-fibrotic microenvironment: In the absence of appropriate regulatory signals - particularly anti-fibrotic miRNA cargo delivered by exosomes - the healing microenvironment favors TGF-β1-driven fibrotic collagen III production over functional collagen I. The result is scar tissue with inferior mechanical properties, not organized tendon matrix.

Each component of the Regenerative Trifecta is selected specifically to address one of these three deficits. The protocol is not a combination of treatments applied simultaneously by coincidence; it is a mechanistically coherent strategy designed to restore the biological conditions that healthy tissue healing requires [3,4].

Soft tissue injury crisis in professional sports

NBA as a Clinical Case Series

The 2025-26 NBA season crystallized what epidemiological data had been accumulating for years: soft tissue injuries - particularly hamstring strains and Achilles tendon ruptures - are occurring at unprecedented rates, eliminating elite athletes at the most critical competitive junctures. Journalistic analyses compiled by sports data reporters confirmed that soft tissue injuries are on the rise leaguewide, a trend playing out in devastating fashion across consecutive playoff cycles [11].

Table 1 summarizes the most clinically significant cases from the 2024-25 and 2025-26 seasons.

**Table 1:** Most clinically significant soft tissue injury cases from the 2024-25 and 2025-26 NBA seasons. NBA, National Basketball Association

Player	Injury/season impact	Outcome	Return status
Jayson Tatum	Ruptured right Achilles, 2024-25 playoffs	Lost 2024-25 Finals; missed 66 regular-season games in 2025-26	Returned 2025-26 (16 regular, 6 playoff games)
Damian Lillard	Torn left Achilles, 2024-25 playoffs	Season-ending; missed entire 2025-26 season	Status uncertain for 2026-27
Tyrese Haliburton	Torn right Achilles, Game 7 Finals 2024-25	Lost Finals; missed entire 2025-26	Recovering for 2026-27
Luka Doncic	Grade 2 left hamstring, April 2026	Season-ending; played only Game 1 of 2026 playoffs	In rehabilitation
Aaron Gordon	Hamstring + calf injuries, 2025-26	Played only 36 games; missed 3 playoff games vs. Minnesota	Limited mobility throughout playoffs
Peyton Watson	Grade 2 right hamstring re-aggravation	Missed entire Nuggets-Timberwolves series	Career trajectory at risk
Donte DiVincenzo	Ruptured Achilles vs. Nuggets, 2025-26 playoffs	Series-ending injury	Recovery timeline unknown

The Nuggets’ experience included Aaron Gordon playing only 36 games due to hamstring and calf issues, and Peyton Watson missing the entire playoff series against Minnesota after re-aggravating a Grade 2 hamstring injury. Both injuries followed the same pattern: initial fibrotic repair, RTS on tissue with inferior mechanical properties, and subsequent re-injury under competitive loading [11].

Perhaps the most medically instructive case is Jayson Tatum, who ruptured his right Achilles in the 2024-25 playoffs and made what commentators described as a 'miraculous recovery' to return for 16 regular season games and 6 playoff games in 2025-26. His compressed recovery timeline - dramatically shorter than historical Achilles rupture outcomes of 9-12 months to competitive return - was aided by aggressive use of advanced regenerative and physical therapy protocols. While Jayson Tatum's case is simply a case report with an *n* = 1, it has the potential to represent the growing integration of orthobiologic strategies at the elite level. 

Pattern Across Professional Sports

The NBA trend mirrors what researchers have documented across the full spectrum of professional sports. Bullock et al. analyzed publicly available injury data from MLB, NBA, NFL, and NHL over 13 seasons (2007-19), documenting a mean of 62.49 injuries per 100 players per season with an increasing temporal trend, particularly for soft tissue pathologies of the lower extremity [1].

In MLB, hamstring and oblique muscle strains dominate the injured list, with average recovery timelines of 18-28 days under standard care (MLB.com). In professional soccer (Premier League), a study of hamstring injuries found an average absence of 18.4 days and a re-injury rate of approximately 12%-18% within the same season. The common biological thread across all professional sports is the same: fibrotic, incomplete healing that produces tissue vulnerable to re-injury under the mechanical demands of elite athletic performance [1,4].

Growing Masters Athlete Population

The professional sports crisis mirrors the injury burden facing masters and recreational athletes. Americans aged 50 and above now participate in sport and recreational fitness at rates previously seen only in younger cohorts. The pickleball phenomenon exemplifies this shift: the sport grew from an estimated 4.8 million players in 2022 to over 13.6 million in 2025, with the largest growth segment being adults over 55 - precisely the demographic with the most compromised intrinsic healing capacity [13].

Lee et al. analyzed national emergency department data from 2004 to 2023 for pickleball-related injuries, confirming that fractures (28.7%) and sprains/strains (24.7%) dominate the injury distribution, with adults aged 60 and older showing the highest proportions of wrist and upper trunk injuries and the greatest rate of floor-impact mechanisms. This population faces what might be termed the *biology double-bind*: they demand the same tissue performance as younger athletes, but operate with healing systems progressively deficient in the key biological resources - MSC numbers, growth factor secretion capacity, and inflammatory resolution mechanisms - that soft tissue healing requires [17,18].

After age 40, MSC numbers in adipose and bone marrow decline measurably, growth factor secretion decreases, and the vascular and paracrine responses that should initiate and terminate the healing cascade become less reliable. The result is slower healing, higher rates of chronic tendinopathy, and a much higher probability of incomplete repair leading to re-injury - precisely the population that stands to gain most from regenerative intervention [10].

R3 Stem Cell and the Regenerative Trifecta

R3 Stem Cell has developed a proprietary, multi-modal regenerative protocol - the Regenerative Trifecta - that combines the three most evidence-supported biological modalities in regenerative sports medicine into a single coordinated treatment strategy. The protocol is not a sequential or additive combination; it is designed so that each component addresses a distinct biological deficit while creating the physiological conditions that enhance the efficacy of the other two. Table 2 summarizes the mechanistic contribution of each component [3,4,6].

**Table 2:** Components of the Regenerative Trifecta. PDGF, platelet-derived growth factor; TGF-β, transforming growth factor-beta; VEGF, vascular endothelial growth factor; EGF, epidermal growth factor; IGF-1, insulin-like growth factor-1; FGF, fibroblast growth factor; UC-MSC, umbilical cord-derived mesenchymal stem cells; RCT, randomized controlled trial

Component	Primary mechanism	Key molecular targets	Evidence level
UC-MSC Cells	Paracrine signaling, immunomodulation, M1→M2 macrophage polarization	Tendon, ligament, cartilage, systemic inflammation	Phase I/II RCTs; multiple systematic reviews and case series
Platelet-Rich Plasma (PRP)	Concentrated growth factor delivery: PDGF, TGF-β, VEGF, EGF, IGF-1, FGF	Fibroblast proliferation, collagen synthesis, angiogenesis	Multiple RCTs; several Cochrane-grade meta-analyses
Exosomes (EVs)	Cell-free extracellular vesicles; miRNA/mRNA cargo; anti-fibrotic cytokine modulation	Tendon-to-bone interface; TGF-β1 fibrosis pathway; collagen I vs. III ratio	Preclinical + systematic review of in vivo studies; emerging translational data

Component 1: UC-MSCs

MSCs derived from human umbilical cord Wharton's jelly represent the most biologically active cellular component of the Trifecta. Caplan and Correa's foundational 2011 paper in Cell Stem Cell described MSCs as site-regulated *drugstores* that, when released from their perivascular location following injury, establish a regenerative microenvironment through the secretion of bioactive molecules and modulation of the local immune response [3].

UC-MSCs offer advantages over some autologous sources, including higher proliferative capacity, robust paracrine activity, and reduced immunogenicity, as demonstrated in comparative studies [19]. Hass et al. compared adult and neonatal tissue-derived MSCs, demonstrating that neonatal-sourced cells exhibit superior multipotency and immune-modulatory properties. In clinical practice, the use of allogeneic UC-MSCs from screened donors provides a consistent, high-potency cell population independent of the patient's age-related MSC decline - a critical advantage for the masters athlete population who most need cellular supplementation [19].

In the context of soft tissue injury, UC-MSC administration addresses Deficit 1 directly: by providing exogenous MSCs to the injury site, the Trifecta compensates for the age-related and injury-related deficiency of endogenous MSCs that would otherwise orchestrate the healing response. Bernardo and Fibbe demonstrated the macrophage polarization capacity of MSCs in detail, confirming their role as principal regulators of the M1-to-M2 transition [9].

Component 2: PRP

PRP delivers an autologous, concentrated payload of growth factors to the injured tissue environment. Activated platelets release PDGF, TGF-β, VEGF, epidermal growth factor (EGF), IGF-1, and fibroblast growth factor (FGF) - molecules that are active across multiple phases of healing: PDGF regulates chemotaxis and angiogenesis during the inflammatory phase; TGF-β promotes collagen synthesis and matrix remodeling; VEGF drives new vessel formation, directly addressing the vascular deficit that underlies delayed tendon and ligament repair [6,20,21].

PRP addresses Deficit 2 of the Trifecta model: inadequate growth factor delivery. By flooding the hypovascular injury site with a concentrated autologous cocktail of precisely the molecules that tissue-resident fibroblasts and tenocytes require to initiate and sustain proliferative healing, PRP overcomes the growth factor insufficiency that prolongs recovery under standard care. PRP also acts as a biological amplifier for UC-MSC activity, providing the substrate that enhances MSC engraftment, survival, and paracrine output at the injection site [6,21].

A critical variable in PRP efficacy is leukocyte content. Leukocyte-poor PRP has demonstrated superior outcomes in several studies of intra-articular indications, reducing the pro-inflammatory leukocyte burden while preserving growth factor payload. Mishra et al. reviewed the sports medicine applications of PRP comprehensively, proposing a classification system and emphasizing that formulation standardization is essential for interpreting clinical data and optimizing outcomes. R3 Stem Cell's protocol incorporates preparation standards optimized for soft tissue injury applications [21].

Component 3: Exosomes (EVs)

Exosomes - nanoscale (30-150 nm) EVs secreted by MSCs - represent the newest and most rapidly evolving component of the Trifecta. Carrying microRNA, messenger RNA, and protein cargo that can reprogram recipient cell gene expression without requiring live cell delivery, exosomes function as cell-free signaling agents that recapitulate many of the therapeutic effects of MSC transplantation with a superior safety and regulatory profile [22,23].

In the context of soft tissue injury, exosome therapy addresses Deficit 3: the pro-fibrotic healing microenvironment. MSC-derived EVs have been shown in multiple preclinical and in vivo studies to reduce TGF-β1-driven fibrosis pathways - the molecular cascade that produces excessive collagen III deposition and disordered matrix architecture. Huang et al. reviewed translational evidence for exosome therapy in sports medicine, confirming that EV-based therapy reduces scar formation and preserves tendon mechanical properties in soft tissue injury models. Lu et al. conducted the most comprehensive systematic review to date of in vivo MSC-EV studies in tendon and ligament repair, identifying 11 studies all of which reported better tendon/ligament repair following MSC-EV treatment, with mechanistic evidence for attenuated inflammatory response and accelerated matrix regeneration [22,23].

Mechanistic synergy of the Trifecta

The Trifecta components interact synergistically rather than additively. UC-MSCs drive the macrophage polarization and paracrine environment that is prerequisite for organized proliferative healing. PRP delivers the growth factor payload that maximizes fibroblast and tenocyte proliferative response within the MSC-conditioned microenvironment. Exosomes modulate the remodeling phase, ensuring that the collagen deposited during proliferation is organized into functional collagen I matrix rather than disorganized scar [4,9,22].

Critically, this sequencing means that the efficacy of each component is enhanced by the others. PRP growth factors improve MSC survival and engraftment; MSC paracrine output creates the anti-inflammatory microenvironment in which EV-driven anti-fibrotic signaling is most effective; and the organized tissue matrix produced by EV modulation provides the structural substrate on which accelerated PRP-driven proliferation can be deposited in a mechanically appropriate architecture [3,4,22].

Clinical evidence: Regenerative biologics and RTS

Overview of the Evidence Base

The clinical evidence base for regenerative biologics in sports medicine has matured substantially over the past decade. While early studies were limited by small sample sizes, heterogeneous formulations, and absence of blinding, the current literature includes multiple Level I randomized controlled trials, high-quality systematic reviews, and several Cochrane-methodology meta-analyses that permit quantitative pooling of RTS outcomes. Table 3 summarizes key clinical findings relevant to the Trifecta's three components [4,24,25].

**Table 3:** Key clinical findings relevant to the Regenerative Trifecta's three components. PRP, platelet-rich plasma; RTS, return to sport; RCT, randomized clinical trial; PT, physical therapy; WOMAC, Western Ontario and McMaster Universities Osteoarthritis Index; VAS, visual analog scale; HA, hyaluronic acid; VISA-A, Victorian Institute of Sport Assessment-Achilles; CI, confidence interval

Study/authors	Injury type	Intervention	Key return-to-sport/outcome finding	Reference
Lu et al. (2021)	Tendon/ligament (systematic review, 11 in vivo studies)	MSC-derived extracellular vesicles	All studies reported better tendon/ligament repair; attenuation of inflammatory response and accelerated matrix regeneration.	[22]
Huang et al. (2024)	Musculoskeletal/soft tissue	Exosome/EV therapy (mini-review)	EV therapy reduces fibrotic scarring; preserves tendon mechanical properties; anti-fibrotic via TGF-β1 pathway downregulation.	[23]
Sheth et al. (2017)	Acute muscle (grade I-II)	PRP vs. standard care (meta-analysis, 5 RCTs, *n* = 268)	Significantly earlier RTS: mean -5.57 days (95% CI -9.57 to -1.58; *P* = 0.006)	[25]
Grassi et al. (2018)	Acute muscle injuries	PRP vs. placebo/PT (meta-analysis, 6 RCTs, *n* = 374)	Mean -7.17 days RTS in PRP group across all studies; effect attenuated in double-blind subgroup	[26]
Desouza and Shetty (2025)	Grade 2 hamstring (*n* = 60 RCT)	PRP + standard rehab vs. standard rehab alone	RTP: 26.4 ± 4.5 vs. 34.2 ± 5.7 days (*P* < 0.001); MRI healing 70% vs. 36.7% at 21 days (*P* = 0.003)	[27]
Krystofiak et al. (2023)	Mixed soft tissue (*n*= 35)	PRP + long-duration ultrasound vs. PRP alone	Combined modality reduced RTS by a mean of 21.33 days vs. PRP monotherapy; all athletes returned to sport.	[28]
Vithran et al. (2023)	Achilles tendinopathy (meta-analysis, 8 RCTs)	PRP vs. placebo	Significant VAS improvement at 12 weeks (*P* < 0.00001); VISA-A and RTS not significantly different overall	[29]
Belk et al. (2020)	Knee OA (18 Level I studies, *n* = 1,608)	PRP vs. hyaluronic acid (meta-analysis)	Mean WOMAC [24] improvement 44.7% (PRP) vs. 12.6% (HA); *P* < 0.01	[30]

PRP in Acute Muscle Injuries: RCT and Meta-analytic Evidence

The most robust clinical evidence for PRP in sports medicine addresses acute muscle injuries - the category most directly relevant to hamstring, quadriceps, and calf pathology in professional athletes. Two major meta-analyses have quantified the RTS benefit of PRP in this context [25,26].

Sheth et al. conducted a systematic review and meta-analysis of five RCTs enrolling 268 athletes with grade 1 or 2 acute muscle strains. Pooled analysis demonstrated a statistically significant earlier RTS in the PRP group (mean difference: -5.57 days; 95% CI: -9.57 to -1.58; *P *= 0.006). The authors noted that subgroup analysis restricted to hamstring injuries alone did not reach significance, suggesting that PRP efficacy may be injury-specific and dependent on the degree of muscle vascularization and innervation at the injury site. The re-injury rate was not significantly different between groups at six months, suggesting that PRP accelerates recovery timeline without compromising tissue durability within this follow-up window [25].

Grassi et al. extended this analysis in a meta-analysis of six RCTs (*n* = 374 patients), reporting a mean RTS difference of -7.17 days favoring PRP. However, the authors classified the overall evidence quality as low to very low by Grading of Recommendations Assessment, Development, and Evaluation (GRADE) criteria due to performance bias [26].

The most recent and methodologically rigorous RCT comes from Desouza and Shetty, who randomized 60 athletes with MRI-confirmed grade 2 hamstring injuries to ultrasound-guided PRP plus standard rehabilitation versus standard rehabilitation alone, with a two-year follow-up. The PRP group demonstrated significantly faster return to play (26.4 ± 4.5 vs. 34.2 ± 5.7 days; *P *< 0.001), superior MRI healing rates at 21 days (70% vs. 36.7%; *P* = 0.003), and a numerically lower re-injury rate (3.3% vs. 16.7%), though the latter did not reach statistical significance. This trial provides the most direct clinical evidence for the PRP component of the Trifecta in the specific injury type - hamstring strain - most commonly responsible for elite athlete elimination in the current professional sports landscape [27].

Krystofiak et al. evaluated 35 athletes with soft tissue injuries treated with combined PRP and long-duration ultrasound (LDU) versus PRP alone. All 35 athletes returned to sport, and the combined modality group demonstrated a mean 21.33-day reduction in RTS time compared to PRP monotherapy - suggesting that even within the PRP modality, combination with adjunct physical medicine modalities produces additive acceleration of recovery. This finding is mechanistically consistent with the Trifecta rationale: each biological and physical modality addresses a distinct aspect of the healing response, and combination produces results neither achieves alone [28].

PRP in Tendinopathy: Evidence, Formulation Factors, and Limitations

The evidence for PRP in chronic tendinopathy, particularly Achilles tendinopathy, is more heterogeneous and requires nuanced interpretation. Vithran et al. conducted a systematic review and meta-analysis of eight RCTs evaluating PRP versus placebo for Achilles tendinopathy, finding a statistically significant improvement in VAS pain scores at 12 weeks (*P *< 0.00001) but non-significant differences in VISA-A functional scores across follow-up time points. The authors concluded that PRP lacks clear superiority over placebo for overall functional outcomes in Achilles tendinopathy, while acknowledging that the heterogeneity of PRP formulations across studies limits the strength of pooled conclusions [29].

This pattern - significant pain reduction with variable functional improvement - reflects the heterogeneity of PRP preparation protocols across clinical studies. Leukocyte content, platelet concentration, activation method, and injection volume all influence the biological activity of PRP and its suitability for different tissue types and healing phases. Everts et al. published the most comprehensive contemporary review of PRP performance parameters, establishing that platelet dosing, leukocyte activity, serotonin effects, and the interaction between PRP and rehabilitation protocols are all determinants of clinical outcome. Standardization of these variables is essential for producing reproducible results and for clinical trial design [6].

For joint osteoarthritis applications, the evidence is more consistently favorable. Belk et al. conducted a meta-analysis of 27 Level I studies (*n *= 2,396 patients) comparing PRP, bone marrow aspirate concentrate (BMAC), and hyaluronic acid for knee osteoarthritis, demonstrating significantly better Western Ontario and McMaster Universities Osteoarthritis Index (WOMAC) (*P *< 0.001), VAS pain (*P *< 0.01), and International Knee Documentation Committee (IKDC) functional scores (*P *< 0.001) in PRP-treated patients compared to HA controls [19,24]. Their earlier 2020 meta-analysis of 18 Level I RCTs (*n* = 1,608 patients) showed mean WOMAC improvement of 44.7% in the PRP group versus 12.6% in the HA group (*P *< 0.01). Leukocyte-poor PRP was associated with superior IKDC outcomes, consistent with the hypothesis that reducing pro-inflammatory leukocyte burden optimizes the growth factor environment at intra-articular injection sites [24,30].

MSC therapy: Structural healing quality

While PRP excels at accelerating the acute healing response through growth factor delivery, MSC therapy addresses the more fundamental question of healing quality. MSC-mediated M1-to-M2 macrophage polarization is the critical determinant of whether injured soft tissue heals with an organized collagen I matrix or fibrotic collagen III scar [3,9,10].

Published comparative analyses support the mechanistic complementarity of MSCs and PRP. Koshy et al. reviewed the evidence for biologic therapies in sports-related tendon and ligament injuries in a Cureus narrative review, concluding that, while PRP demonstrates sustained improvements in pain and function for chronic tendinopathies, MSCs show particular promise in enhancing graft integrity following ligament reconstruction and promoting more organized tissue architecture. The review identified variability in preparation protocols and limited long-term RCTs as the primary constraints on definitive conclusions, consistent with the broader literature [4].

Yin et al. established an important mechanistic foundation for MSC therapy in tendon healing, demonstrating that the physical alignment of the healing microenvironment - mimicked by aligned electrospun nanofibers in their study - drives tendon stem cell differentiation toward organized tendon-specific gene expression rather than osteogenic pathways. This work supports the concept that the biological microenvironment established by MSC paracrine signaling, not simply MSC differentiation per se, determines the quality of tendon repair [7].

Du et al. reviewed advanced regenerative therapies for joint-related sports injuries, emphasizing the convergence of cellular therapies, growth factor delivery, and extracellular matrix engineering as the frontier of orthobiologic sports medicine. The authors' synthesis is consistent with the Trifecta model: no single biological modality addresses all three deficits of soft tissue repair, and multi-modal combination is both mechanistically justified and increasingly supported by early clinical evidence [5].

EVs: Emerging translational evidence

The third component of the Trifecta represents the newest and most rapidly evolving frontier of regenerative sports medicine. MSC-derived EVs combine the therapeutic versatility of MSC paracrine signaling with a cell-free delivery platform that circumvents the logistical, regulatory, and viability constraints of live cell therapy [22,23].

Lu et al. conducted the most rigorous systematic review of in vivo MSC-EV studies in tendon and ligament repair, identifying 11 studies from PubMed, Web of Science, Cochrane Library, and Embase that met inclusion criteria (in vivo models with control groups). All 11 studies reported better tendon and ligament repair in MSC-EV-treated animals. The consistent mechanistic findings across studies included attenuation of the initial inflammatory response, accelerated matrix regeneration, and reduced fibrous adhesion formation. Biomechanical parameters were reported in 8 of the 11 studies, with evidence linking EV treatment to measurable improvements in tendon tensile strength - the functional endpoint most directly relevant to RTS durability [22].

Huang et al. synthesized the translational evidence for exosome therapy in sports medicine specifically, reviewing the cargo composition of MSC-derived EVs, their biodistribution at injury sites, and their anti-fibrotic mechanism of action. The authors identified miRNA species including miR-29, miR-140, and miR-222 as key EV cargo molecules that downregulate TGF-β1 signaling and promote organized collagen I deposition - directly addressing the pro-fibrotic microenvironment that represents the third biological deficit in the Trifecta model [23].

One needs to be careful not to over estimate the potential of exosomes for healing injury by themselves, and assume they could replace MSCs. Exosomes cannot replicate core MSC function because they are a static, acellular product, while MSCs are living cells that sense and adapt. Exosomes carry a fixed snapshot of signaling molecules captured at the time of manufacture - they cannot adjust their cargo in response to the body's changing needs once administered. MSCs, by contrast, continuously read their surrounding tissue environment and adjust what they secrete in real time, a process known as licensing.

MSCs also perform functions exosomes are structurally incapable of: they actively migrate toward injury sites (homing), transfer whole functional mitochondria to damaged cells to restore cellular energy production, and retain the potential to directly differentiate into tissue-specific cell types like cartilage or bone. Exosomes offer real advantages in stability, dosing, and safety, but they represent only the paracrine signaling output of the MSC - not the living, responsive, multi-functional cell itself. This is why exosomes complement the Regenerative Trifecta rather than replace the MSC component.

It is important to acknowledge that the clinical evidence for exosome therapy in human sports injuries remains at an earlier stage than that for PRP or MSC therapy. The majority of mechanistic studies are preclinical, and while systematic reviews of in vivo animal models provide strong translational rationale, phase I/II clinical trials in human soft tissue injury specifically are still emerging. The Trifecta protocol incorporates exosome therapy based on this strong mechanistic and in vivo rationale, with the recognition that clinical evidence is accumulating rapidly and the next several years are likely to produce the first controlled human trials of sufficient power to quantify the specific contribution of EV therapy to soft tissue healing outcomes [22,23].

Multi-modal Combination Therapy: The Rationale for the Trifecta

The case for combining PRP, UC-MSCs, and exosomes in a single protocol rests on three pillars: distinct mechanistic complementarity, preclinical evidence of synergistic biological effects, and early clinical data suggesting that multi-modal protocols outperform single-modality interventions [4,28].

From a mechanistic standpoint, the three components are temporally as well as functionally complementary. PRP growth factors are most active in the inflammatory-to-proliferative transition (days 3-14), driving fibroblast recruitment and early collagen synthesis. UC-MSCs provide the paracrine immunomodulatory signals most critical in the early inflammatory phase (days 1-7) and early proliferative phase, ensuring that PRP-driven proliferation occurs in an organized rather than fibrotic microenvironment. Exosomes modulate the remodeling phase (weeks 3-52), refining the collagen architecture of the healed tissue toward functional matrix rather than scar [3,9,22].

Krystofiak et al. provided the most direct clinical evidence supporting combination superiority in soft tissue injury. The combination of PRP plus LDU reduced RTS by 21.33 days compared with PRP alone - a magnitude of effect substantially greater than the 5.57- to 7.17-day advantage observed with PRP over standard care alone. These findings suggest that combination modalities may accelerate recovery through the additive effects of multiple biological signals [28].

The practical clinical implication is that patients receiving the Trifecta are not simply getting three treatments at once; they are receiving a coordinated biological program that addresses every phase of the healing cascade simultaneously, converting the injured microenvironment from one that produces fibrotic scar to one that produces organized, functional soft tissue [3,4].

Expanding at-risk athlete population

Masters Athlete and the Biological Double Bind

Americans aged 50 and above now represent one of the fastest-growing segments of the active sports population, driven by the explosive growth of pickleball, cycling, recreational running, and fitness sports. Lee et al. documented the surge in pickleball-related emergency department presentations using the National Electronic Injury Surveillance System, confirming that older adults (≥60 years) bear the greatest burden of serious injuries, with fractures representing 32.8% of injury diagnoses in this age group [18].

This demographic faces a unique biological situation. The same tissue-level deficit that drives poor healing in professional athletes - vascular insufficiency, macrophage polarization failure, growth factor depletion - is amplified in the masters athlete by age-related changes that compound every aspect of the healing response. MSC numbers and paracrine potency decline measurably after age 40. Growth factor secretion from platelets and tissue-resident fibroblasts decreases. The inflammatory resolution mechanisms that should terminate the healing response and initiate tissue remodeling become less reliable, contributing to the chronic low-grade inflammation sometimes termed *inflammaging* that keeps the microenvironment in a persistently pro-inflammatory state [10,19].

The result is that the same hamstring strain that might sideline a professional athlete for 18-26 days under standard care may keep a masters recreational athlete symptomatic for 6-12 weeks - and may heal incompletely even then. A masters athlete with an Achilles tendinopathy who receives the Trifecta protocol may not simply be recovering faster; they may be achieving a level of tissue quality that is biologically unattainable through passive standard care. This is the clinical case that converts recreational athletes into advocates: not simply an accelerated timeline, but a qualitatively superior result [4,19].

Closing the Knowledge Gap

The majority of recreational and masters athletes remain entirely unaware that the regenerative treatments used by elite professional athletes - historically accessed through international stem cell centers in Panama, Japan, or Germany - are now available in the United States through R3 Stem Cell's network of affiliated providers, operating within established FDA regulatory frameworks for same-day surgical procedures. The knowledge gap between what elite athletes access and what recreational athletes know is available represents the single most impactful area where R3 can drive patient outcomes at scale. 

Athletes who understand their biology - who recognize that their 'healed' hamstring has repaired fibrotically and remains at elevated re-injury risk, and that the Trifecta can address that biology specifically - become active participants in their own regenerative care rather than passive recipients of symptom management. R3 Stem Cell's clinical and educational mission is to bridge this knowledge gap, making evidence-based regenerative biologics accessible to any athlete, professional, masters, or recreational, who wants to recover faster, heal more completely, and return to the sport they love.

Discussion

The convergence of four trends creates both urgency and opportunity for regenerative sports medicine: (1) a documented and worsening epidemic of soft tissue injuries at the professional level; (2) a massive and growing population of masters and recreational athletes with inferior healing biology and limited awareness of regenerative options; (3) a rapidly maturing evidence base confirming that biologics reduce RTS timelines and improve healing quality; and (4) increasing accessibility of these treatments through providers like R3 Stem Cell operating within the United States under established regulatory frameworks [1,4,18].

PRP has been shown to produce a clinically meaningful acceleration of RTS in acute muscle injuries, with consistent point estimates ranging from 5.57 to 7.17 days across independent meta-analyses. In the specific clinical scenario most relevant to professional sports - grade 2 hamstring injury - the most recent RCT demonstrates a 7.8-day absolute reduction in return-to-play time, representing a 23% compression of the recovery timeline. In the context of NBA playoffs, where the competitive value of even a single game can define a season, this magnitude of effect is transformative [25-27].

The mechanistic case for UC-MSC therapy rests on a foundation of high-quality basic science from Cell Stem Cell, Nature Reviews Immunology, and Journal of Cellular Physiology, establishing the paracrine immunomodulatory role of MSCs with high confidence. Translation of this mechanistic evidence to human soft tissue injury outcomes requires the larger clinical trial infrastructure that the field is currently building. The clinical evidence for EV therapy is at an earlier stage but is advancing rapidly - the 11-study systematic review by Lu et al. provides a systematic evidence base for preclinical efficacy that is unusually consistent in directionality [2,3,9,10,22].

The challenge facing the field is not primarily scientific uncertainty-the mechanisms are increasingly well characterized-but rather the translation of this understanding into clinical practice, alongside ongoing questions about optimal dosing, timing, patient selection, and long-term outcomes [4]. The majority of athletes, sports medicine physicians, and athletic trainers at every level continue to regard rest, ice, and physical therapy as the ceiling of what regenerative medicine can offer injured tendons and ligaments. Changing this belief, through rigorous outcomes data, patient-reported results, and the growing roster of elite athletes who have experienced regenerative biologics firsthand, is the educational and clinical imperative for the field over the next decade [4].

The Regenerative Trifecta protocol is positioned at the intersection of mechanistic completeness and clinical accessibility: it addresses all three biological deficits of soft tissue repair (paracrine signaling, growth factor delivery, and anti-fibrotic modulation); it combines the three modalities with the strongest evidence base in regenerative sports medicine; and it is available today, in the United States, through a network of affiliated providers operating under consistent clinical protocols developed and refined by R3 Stem Cell. 

Future research priorities include: (1) prospective outcomes registry for Trifecta-treated athletes across R3's provider network, enabling the generation of real-world evidence at scale; (2) imaging biomarker studies using quantitative MRI and elastography to characterize tissue quality in Trifecta-treated versus standard care patients; (3) re-injury rate tracking with minimum 24-month follow-up; and (4) subgroup analyses identifying which athlete characteristics (age, injury type, and chronicity) most strongly predict response to each Trifecta component. 

A candid assessment of the current evidence base identifies several important limitations that should inform clinical interpretation. For PRP in acute muscle injuries, the GRADE quality of evidence is low to very low in aggregate meta-analyses, driven by performance bias from lack of patient blinding in the majority of available RCTs. For MSC therapy in soft tissue injury specifically, large-scale RCT data in human patients is limited, with most mechanistic evidence derived from in vitro studies and animal models. For exosome therapy, human clinical trial data remains sparse [4,26].

These limitations reflect the rapidly evolving nature of regenerative sports medicine rather than fundamental scientific uncertainty about the mechanisms involved. The biological rationale for each Trifecta component is well-established in peer-reviewed literature from high-impact journals. The clinical evidence is accumulating in the direction consistent with mechanistic predictions. What the field lacks is the large-scale, double-blind, long-term RCT infrastructure that has been built up over decades for pharmacological sports medicine interventions - infrastructure that will take time to develop for these newer biological therapies. 

Future directions most critical for the field include: standardization of PRP preparation protocols to enable meaningful cross-study comparison; phase II/III RCTs with adequate power and blinding for MSC and EV therapy in human soft tissue injury; long-term follow-up studies (minimum 24 months) examining re-injury rates and tissue quality outcomes; and biomarker studies identifying the patient populations most likely to benefit from each component of the Trifecta [4,6].

## Conclusions

Soft tissue injuries represent the dominant unsolved problem in athletic medicine. A worsening epidemic of hamstring, Achilles, and ligament injuries across professional leagues - documented most recently in the 2025-2026 NBA season - reflects the fundamental inadequacy of standard care for a biology that requires active regenerative intervention, not passive symptom management. The same biological failure affects millions of masters and recreational athletes who remain largely unaware that effective regenerative options exist.

Taken together, the evidence reviewed in this paper points to a clear conclusion: each component of the Trifecta addresses a distinct piece of the healing puzzle that the others cannot fully replicate on their own. PRP reliably accelerates the acute phase of muscle and tendon repair, meaningfully shortening the timeline back to competition. UC-MSC therapy supplies the mechanistic foundation for healing quality, driving the shift toward organized, functional tissue rather than disorganized scar. Exosome therapy targets the pro-fibrotic signaling that standard care never addresses, with translational findings that point consistently in the same direction across the published literature. No single modality resolves all three of the biological deficits that determine whether an injury heals functionally or fibrotically - which is the central rationale for combining them into one coordinated protocol. 

The vision for patients with soft tissue injuries is straightforward: recovery should mean more than symptom relief and a return to activity on tissue that remains structurally compromised. Whether the patient is an elite athlete racing back for a playoff run or a weekend recreational athlete who simply wants to move without pain, the same underlying biology applies, and the same regenerative tools should be available to both. 

The Regenerative Trifecta is the answer to a simple question: what would care look like if soft tissue injury were treated as a biological problem to be solved, rather than an inconvenience to be managed? As discussed, current evidence exists for the therapy; however, expanded outcomes registries, imaging biomarker studies, and long-term follow-up data will help to elucidate dosing, timing, and RTS improvements.
